# Supplementary material for: Diamond with Sp2-Sp3 composite phase for thermometry at Millikelvin temperatures
Source: Nat Commun. 2024 May 8;15:3871. doi: 10.1038/s41467-024-48137-z (PMC11079005; doi:10.1038/s41467-024-48137-z)
Supplement: Supplementary file 1 — Supporting Information [file 41467_2024_48137_MOESM1_ESM.pdf]

Supplementary figures and tables

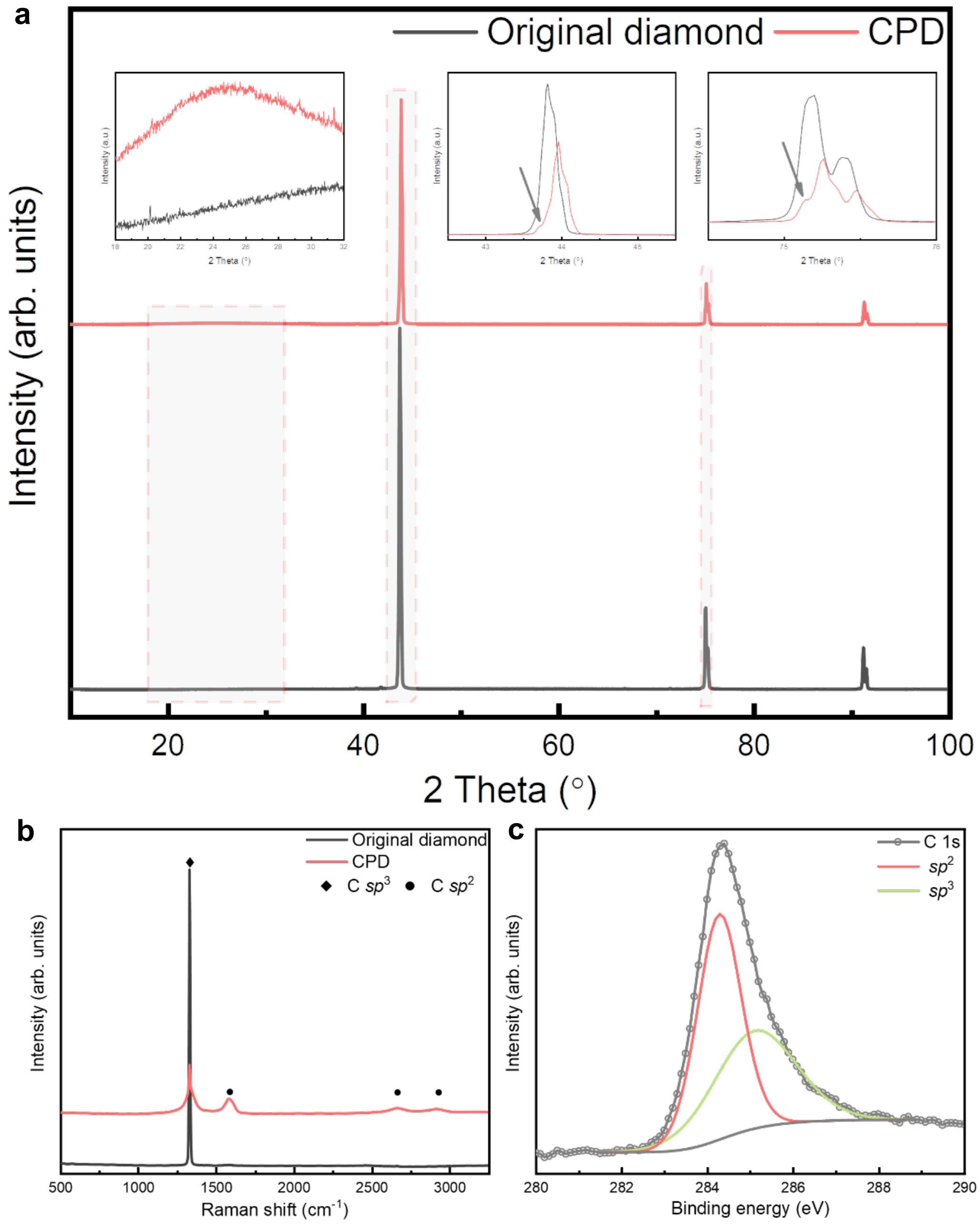

Supplementary Fig. 1 Structural characterization of the CPD. a X-ray diffraction (XRD) patterns of the original diamond and the CPD. Left inset: the magnified sections of the XRD patterns for the CPD within the 18º-32º two-theta range show a weak broadened peak at approximately 2θ = 26º. This peak can be attributed to the (002) reflection of carbon atoms in a hexagonal crystalline arrangement, suggesting the existence of an amorphous carbon structure.

Middle and right insets: the (111) and (220) peaks of CPD display a minor shift of ~0.1º, accompanied by the emergence of shoulder peaks on the left (gray arrows). This indicates a change in the lattice constant of the sample and suggests the possibility of lattice distortion at the nanometer scale. Similar phenomena have been reported for a nanotwinned diamond[1].**b** X-ray photoelectron spectra of the C 1s region of the CPD. The deconvoluted peaks at 284.3 and 285.1 eV correspond to sp^2-hybridized carbon bonds and sp^3-hybridized carbon bonds, respectively. **c** Raman spectra of the CPD. The sharp peak at 1332 cm^{-1}, representing the first-order Raman spectrum line of diamond, corresponds to the symmetric stretching vibrations of sp^3-hybridized carbon atoms within the diamond lattice. The original diamond has a diamond characteristic peak width at the full width at half maximum (FWHM) of 8.3 cm^{-1}. In comparison, the peak of the CPD is broadened to 31.4 cm^{-1}, which is also considered a manifestation of the lattice distortion, splitting, and rearrangement of diamond crystals. The G band at 1583 cm^{-1} is a characteristic band of the sp^2-hybridized carbon phase, with an FWHM of 120 cm^{-1}, which also reflects the presence of an sp^2-hybridized carbon phase within the CPD. The peak at 2915 cm^{-1} is referred to as the D+G peak, which arises from inter-valley scattering of a phonon near the K point, in-valley scattering of a phonon near the K' point, and the inter-valley scattering of a defect. This peak is related to the degree of disorder in the sp^2 carbon[2,3], its appearance indicates the occurrence of a significant number of sp^2/sp^3 bond transitions within the CPD crystal.

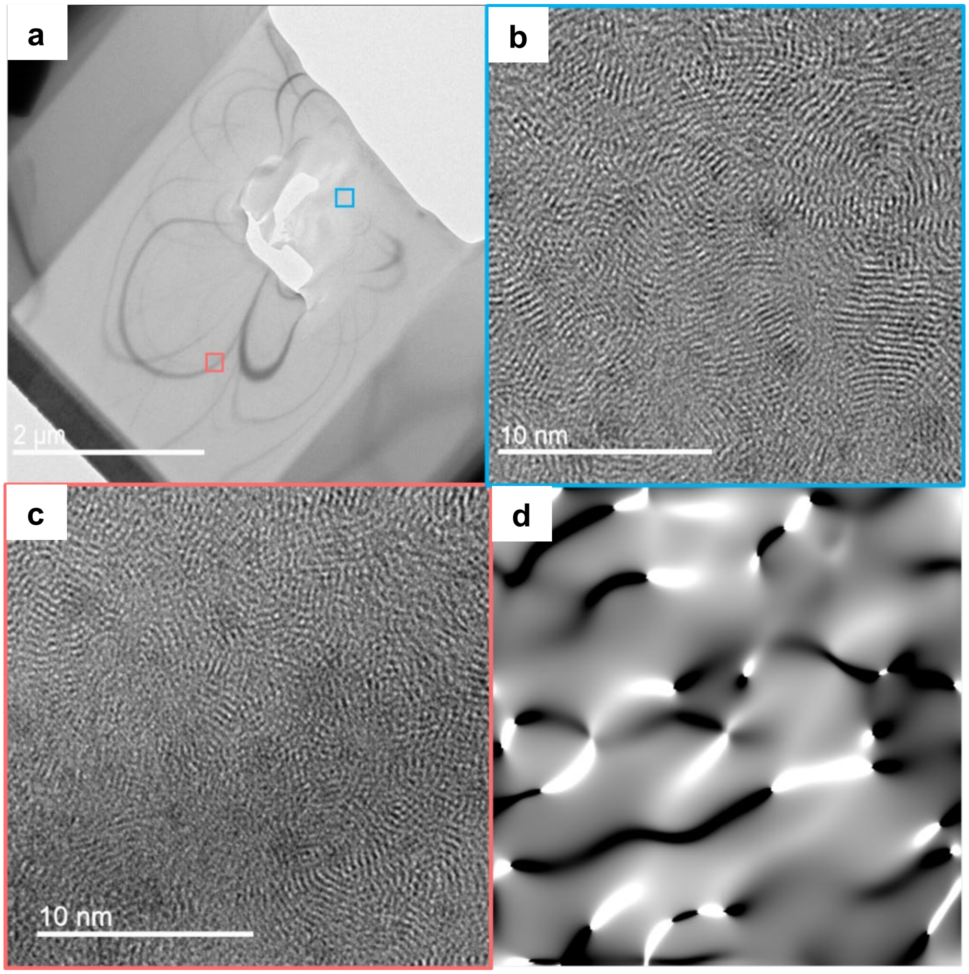

Supplementary Fig. 2 Uniform distribution of nano dual-phase structure at the micrometer scale. a 8000 K magnification of the thin area of the CPD sample prepared with focused-ion beam (FIB) technology with a size of approximately 2×2 μm^2. **b** and **c** High-resolution transmission electron microscopy (HRTEM) images of the selected region marked in the FIB sample showing the repeatable intermittent periodic distribution of the nano dual-phase structure. **d** Geometric phase analysis for background-filtered image (Fig.1b) showing the stress field of the nano dual-phase structure, where tension (white) and compression stress (black) alternately emerge and meet, corresponding to diamond and graphite as well as amorphous carbon transition region.

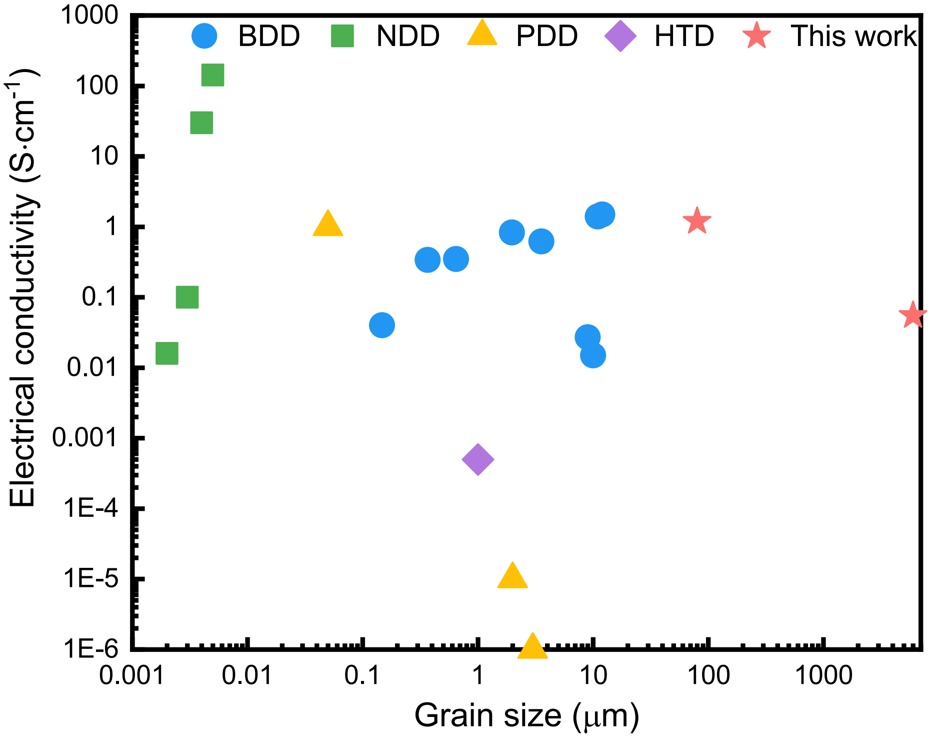

Supplementary Fig. 3 Comparison of the conductivity of the CPD with those of other conductive diamonds: boron-doped diamond (BDD) [4-6], nitrogen-doped diamond (NDD) [7,8], phosphorus-doped diamond (PDD) [9,10]and hydrogen-terminated diamond (HTD) [11]. In single-crystal diamond, the presence of numerous strongly bonded carbon atoms (σ-bonds) results in extremely high activation energy, making it challenging to form an n-type diamond through nitrogen or phosphorus doping[7,12,13]. Therefore, diamond doping is usually conducted using the smallest possible grain size. In this study, the conductivity of the CPD powder with a particle size of 80 μm was found to reached 1.2 $S \cdot cm^{-1}$, while the conductivity of the single-crystal diamond with dimensions of $5 \times 5 \times 1$ mm^3 was measured as 0.05 $S \cdot cm^{-1}$.

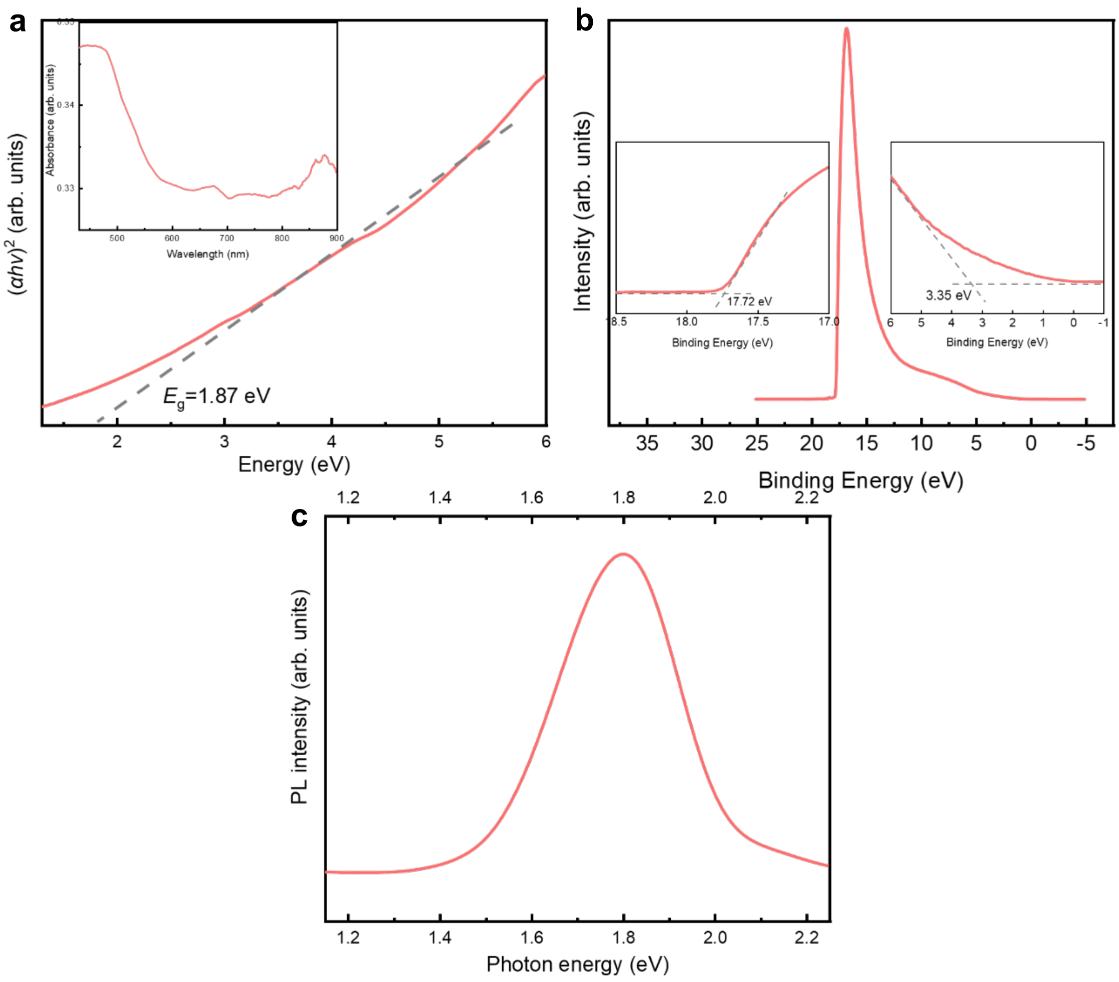

Supplementary Fig. 4 Spectroscopic analysis of energy band information of CPD: a UV-Vis spectrum. Insert: The UV-Vis plots were processed using the Tauc-plot method[14]. The calculated band gap of CPD was 1.87 eV. **b** UPS spectrum. **c** PL spectrum.

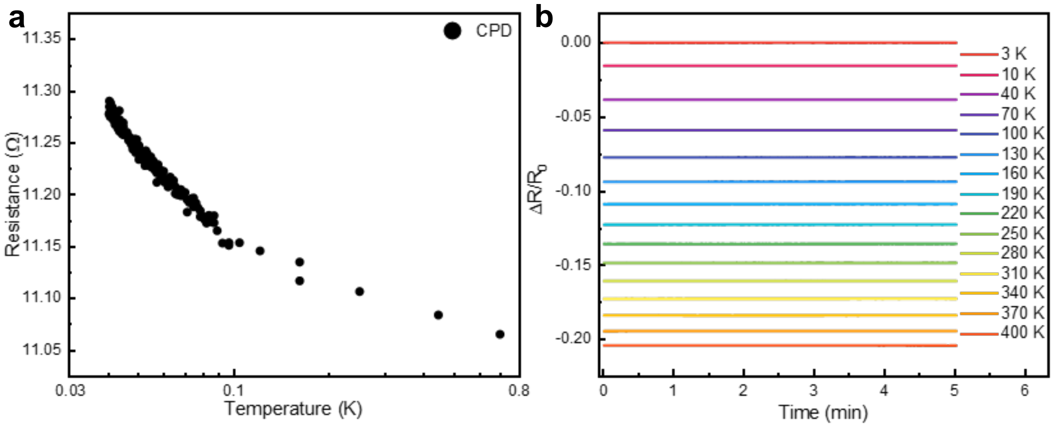

Supplementary Fig. 5 Performance of the temperature sensing characteristics of CPD. a Resistance changes of CPD from 40 to 800 mK. **b** Change rates of resistance response over time at different temperatures. Here, R_0 is the average resistance measured at 3 K.

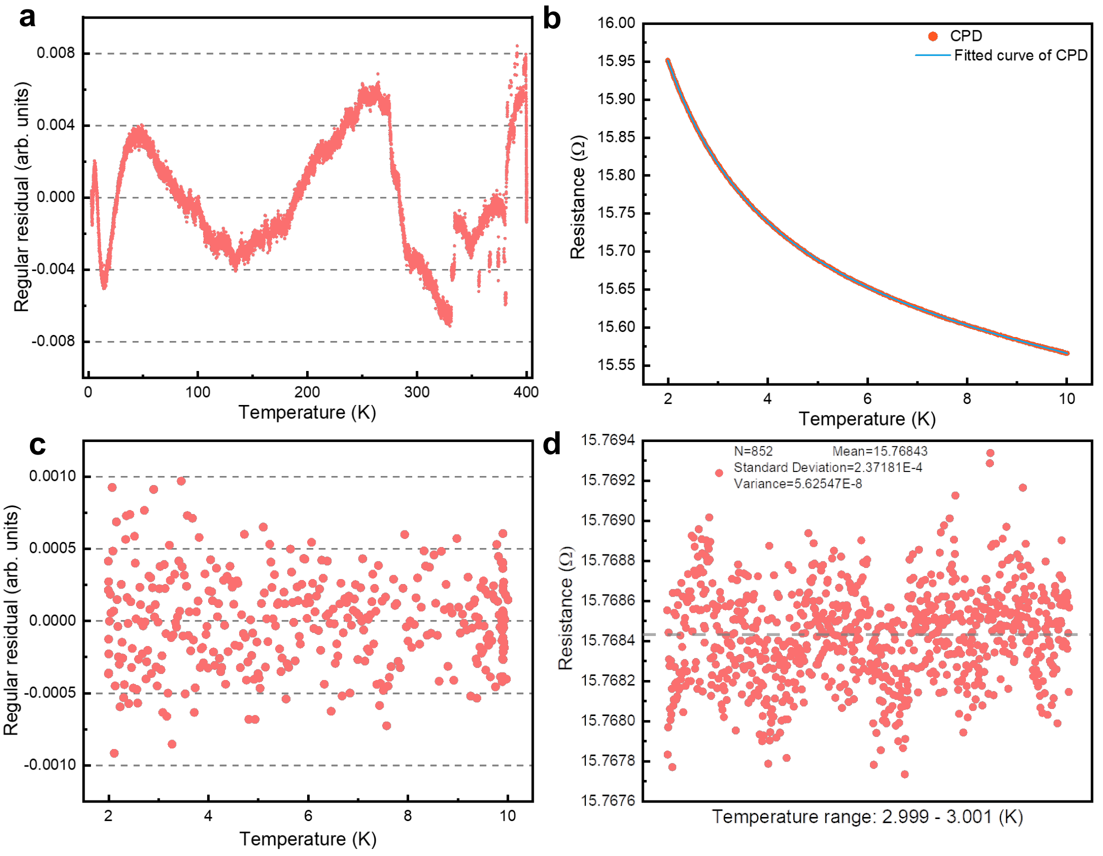

Supplementary Fig. 6 Statistical analysis of the resistance discreteness of the CPD. a The regular residuals between the data fitted by the Expdec3 function and the measured data points. **b** The results of the seventh-order polynomial fitting for the data measured in the temperature range of 2 - 10 K. Red line: experimental data points; blue line: seventh-order polynomial fitting curve. Inset table: the fitting curve parameters and associated statistical errors (R^2). **c** The corresponding regular residual distribution to b. **d** Statistical analysis of the distribution of the resistance values of the CPD obtained at ~3 K. During the dynamic response test, the resistance readings in the 2.999 < T <3.001 K interval were recorded and compared with the reference value obtained from the Cernox™ cryogenic temperature sensor. The resistance values were analyzed to calculate the confidence interval, variance, and standard deviation, as shown in Supplementary Table 1.

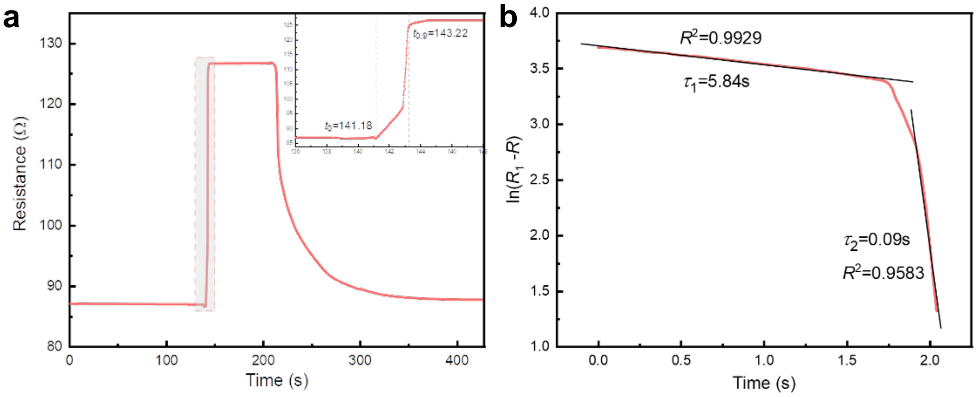

Supplementary Fig. 7 The response time of CPD during rapid temperature changes. **a** Response time of CPD when cooling from room temperature to liquid nitrogen temperature. **b** The calculated thermal time constant of CPD.

The thermal time constant (TTC) is a characteristic constant for the response time of thermistor material. The heat balance equation for an isotropic solid without internal heat sources can be represented as[15]:

$$-\rho \cdot C_p \cdot V dT = \alpha \cdot A \cdot (T - T_1) dt \tag{1}$$

As the direct measurement data in this paper is resistance, and resistance corresponds directly to temperature, all parameters related to T in the above equation have been replaced with R:

$$-\rho \cdot C_p \cdot V dR = \alpha \cdot A \cdot (R - R_1) dt \tag{2}$$

In Eq. (2), A represents the sample's surface area, ρ is the sample's density, Cp and V are the specific heat capacity and volume of the sample, respectively. α is the heat transfer coefficient between the sample and the surrounding medium, and it can be influenced by various factors, including the shape and size of the sample, as well as the method of contacting with the surrounding medium, the form and flow rate of the medium, and more.

When the sample is moved from air to liquid nitrogen, the resistance changes from R_0 to R_1. The relationship between t and R at any given moment during this process can be obtained by integrating equation (2), resulting in:

$$\frac{R - R_1}{R_1 - R_0} = -e^{-\frac{t}{\tau}} \tag{3}$$

Where τ is the thermal time constant,

$$\tau = \frac{\rho C_{\mathrm{p}} V}{\alpha A} \tag{4}$$

Eq. (3) can be further written as:

$$R_1 - R = (R_1 - R_0)e^{-\frac{t}{\tau}} \tag{5}$$

Taking the natural logarithm of both sides of Eq. (5), we can further express it as:

$$\ln(R_1 - R) = \ln(R_1 - R_0) - \frac{t}{\tau} \tag{6}$$

Clearly, plotting $\ln(R_1 - R)$ against t results in a linear relationship, where the slope of the line corresponds to $-\frac{1}{\tau}$, then getting the value of τ. As shown in Supplementary Fig. 6b, the curve is not a single line; instead, it is composed of two linear segments, which is due to the reasons mentioned earlier. We conducted separate curve fitting for the two segments, yielding τ_1 and τ_2. τ_1 is 5.84 seconds, and τ_2 is 0.09 s. τ_1 is situated within the region significantly affected by the intermediate medium (water vapor), leading to a substantial overestimation of its value. In contrast, $t_{0.26}$ can still be reasonably regarded as the starting point of the step-change temperature. Therefore, τ_2 holds greater reference value.

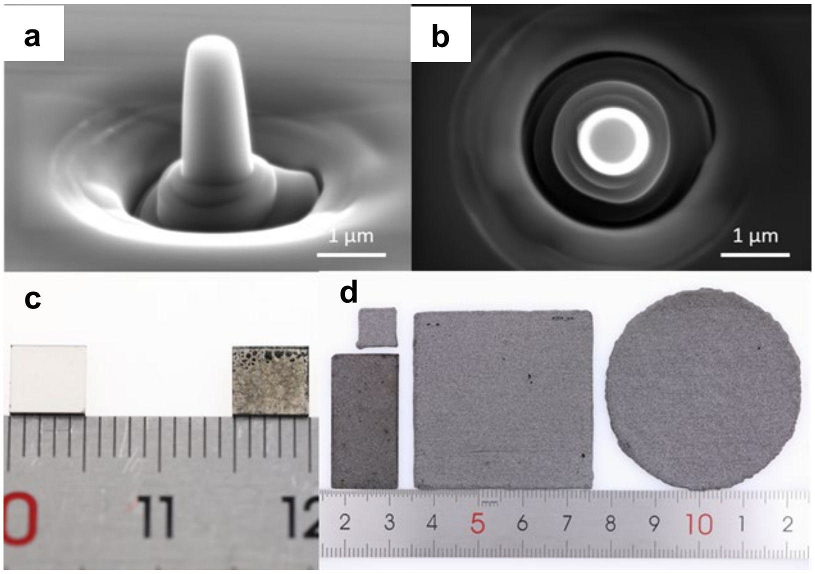

Supplementary Fig. 8 Images of actual CPD samples. a and **b** A pillar-like CPD probe, Ø=1 µm, prepared via FIB. **c** A CPD sample prepared from a (110) single-crystal diamond, with dimensions of 5 × 5 × 1 mm^3. **d** 3D-printed CPD samples, printed from diamond powder with a particle size of 80 µm.

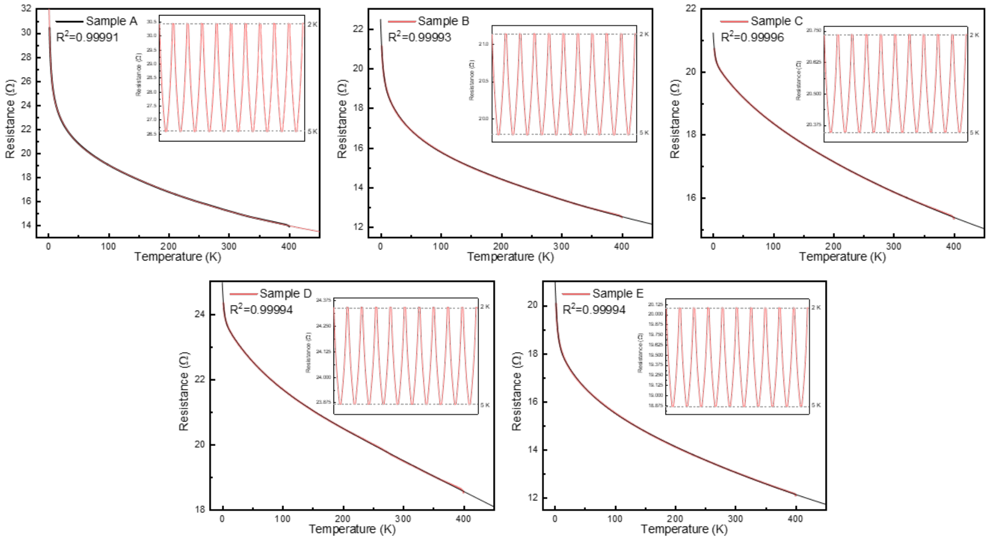

Supplementary Fig. 9. *R*-*T* curves of CPD samples with different sizes: sample A (8×4 mm^2), sample B (4×4 mm^2), sample C (8×5 mm^2), sample D (10×5 mm^2), and sample E (5×5 mm^2). All samples showed NTC characteristics. There are slight differences in resistance values among samples of different sizes. On one hand, the differences in resistance are expected due to the varying sizes of the samples. On the other hand, manual operations inherently introduce various unavoidable sources of error, so these variations are understandable.

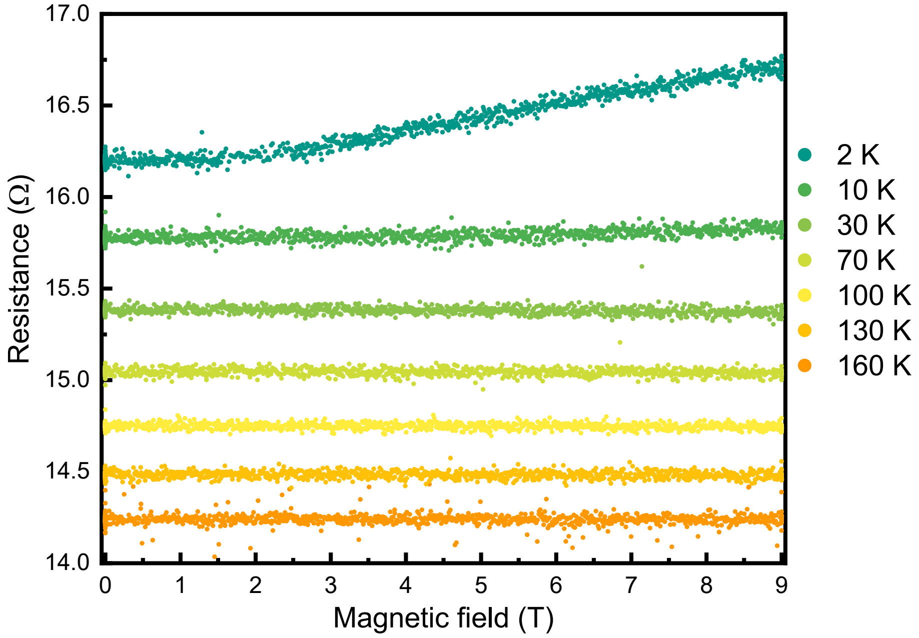

Supplementary Fig. 10 Stability of the CPD under magnetic fields. The resistance of the CPD with increasing applied magnetic fields from 0 to 9 T at different cryogenic temperatures. At temperatures above 10 K, the CPD was almost insensitive to external magnetic fields. At 2 K, the resistance of the CPD shifted when the magnetic field exceeded 2 T, and the CPD exhibited a linear magnetoresistance feature.

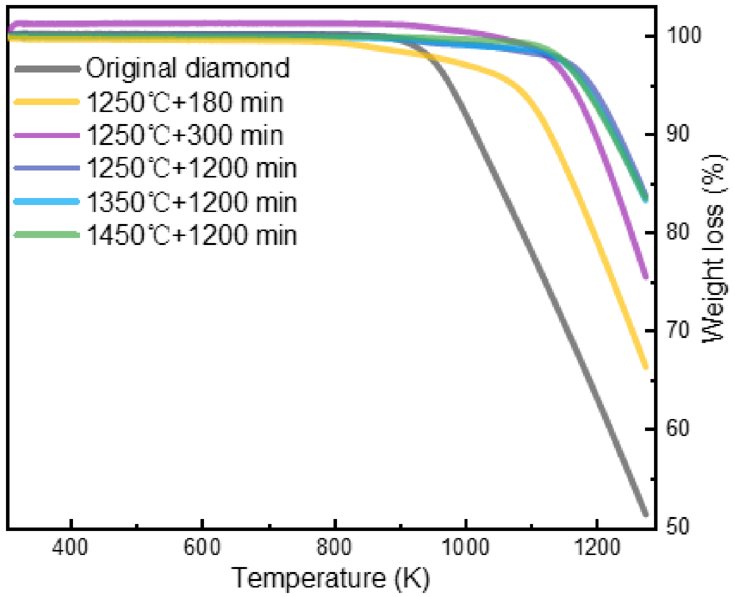

Supplementary Fig. 11 Effect of heat treatment conditions on the thermal stability of the CPD. Thermogravimetric curves with different heat treatment processes. With an increase in the heat treatment time, the onset thermal oxidation temperature of the CPD increased significantly, and the maximum value was reached at 1200 min. The effect of heat treatment temperature on the thermal stability of the CPD was not significant.

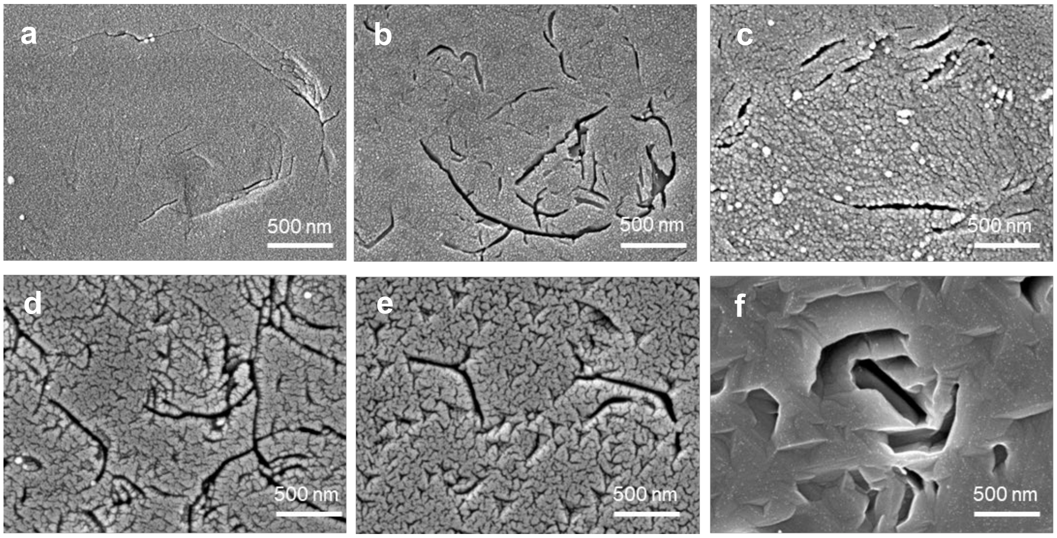

Supplementary Fig. 12. **Oxidation of original diamond at different temperatures. a** 700 °C, 3min. **b** 900 °C, 3 min. **c** 1000 °C, 3 min. **d** 1000 °C, 10 min. **e** 1000 °C, 30 min. **f** 1100 °C, 3 min.

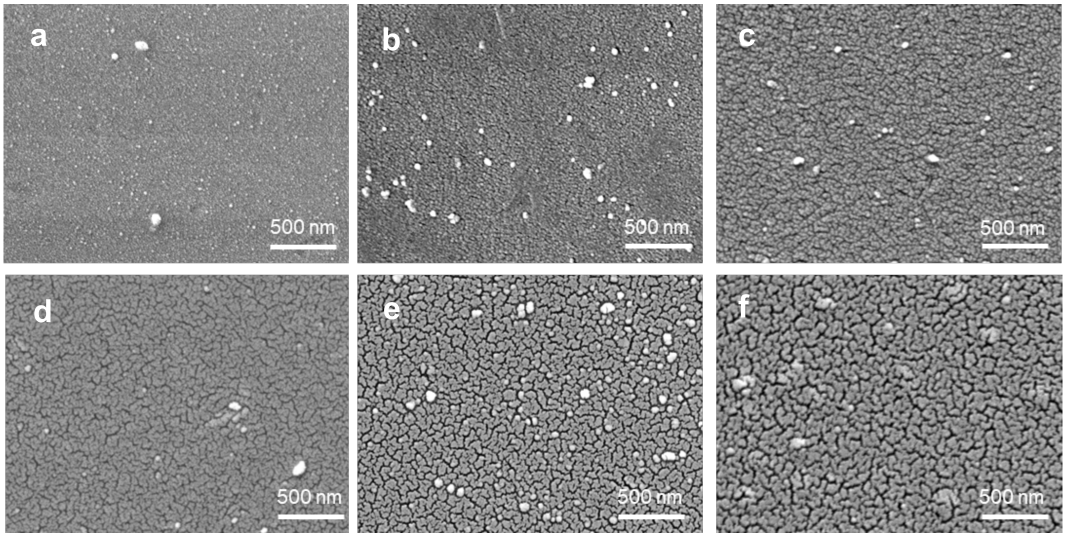

Supplementary Fig. 13. Oxidation of CPD at different temperatures. a 700 °C, 3min. **b** 900 °C, 3 min. **c** 1000 °C, 3 min. **d** 1000 °C, 10 min. **e** 1000 °C, 30 min. **f** 1100 °C, 3 min.

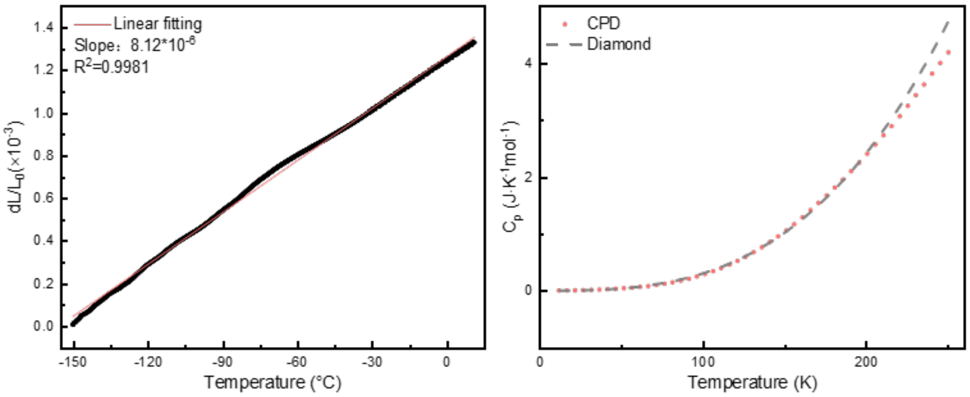

Supplementary Fig. 14. **Thermal properties of CPD. a** The thermal expansion coefficient of CPD. **b** The heat capacity of CPD. According to the Debye model[16], as the temperature approaches absolute zero, the heat capacity of CPD also approaches zero. Based on experimental values, at 25 K, CPD's heat capacity is already less than 0.01 $J{\cdot}K^{-1}mol^{-1}$. CPD's low heat capacity and low resistance can effectively reduce the self-heating effect during temperature measurements.

Supplementary Table 1 Descriptive statistics of the collected resistance data points when the CPD was tested at 3 K.

Interval	N total	Mean	Standard Deviation	Variance	Lower 95% CI of Mean	Upper 95% CI of Mean
$2.999 \le T < 3$	401	15.76847	2.4066×10^{-4}	5.79171×10^{-8}	15.76844	15.76849
$3 \le T < 3.001$	451	15.76839	2.28934×10^{-4}	5.2411×10^{-8}	15.76837	15.76841

Supplementary Table 2 Cryogenic temperature sensor performance indicators. Except for the CPD data, all data were sourced from the public disclosures of Lake Shore Cryotronics, Inc.

Cryogenic temperature sensor	Resolution at 2 K (k)	Lower temperature limit (k)	Upper temperature limit (k)	Sensor size (mm^3)	Resistance at 2 K (Ω)	Resistance shift rate at 2 K and 8 T (%)
CPD	0.001	0.001	500	2×10^{-3}	15.95	2.6
Cernox™	0.005	0.1	420	0.411	413.26	3.1
Interchangeable Rox™	0.005	0.05	40	1.2	1726	7.9
Germanium	0.006	0.05	100	72.9	19.32	60
Silicon Diodes	0.12	1.4	500	0.031	*	*
Platinum	1.3	14	873	24.115	*	*

References

1. Huang, Q. *et al.* Nanotwinned diamond with unprecedented hardness and stability. *Nature* 510, 250-253 (2014).

2. Saito, R., Hofmann, M., Dresselhaus, G., Jorio, A. & Dresselhaus, M. S. Raman spectroscopy of graphene and carbon nanotubes. *Adv. Phys.* 60, 413-550 (2011).

3. Saito, R. *et al.* Probing phonon dispersion relations of graphite by double resonance Raman Scattering. *Phys. Rev. Lett.* 88, 4 (2002).

4. Fischer, A. E. & Swain, G. M. Preparation and characterization of boron-doped diamond powder. *J. Electrochem. Soc.* 152, B369 (2005).

5. Kondo, T. Conductive boron-doped diamond powder/nanoparticles for electrochemical applications. *Chem. Lett.* 50, 733-741 (2021).

6. Kondo, T. *et al.* Boron-doped diamond powders for aqueous supercapacitors with high energy and high power density. *J. Electrochem. Soc.* 166, A1425-A1431 (2019).

7. Zkria, A. *et al.* Correlated electrical conductivities to chemical configurations of nitrogenated nanocrystalline diamond Films. *Nanomaterials* 12, 1-12 (2022).

8. Bhattacharyya, S. *et al.* Synthesis and characterization of highly-conducting nitrogen-doped ultrananocrystalline diamond films. *Appl. Phys. Lett.* 79, 1441-1443 (2001).

9. Kato, H. *et al.* Heavily phosphorus-doped nano-crystalline diamond electrode for thermionic emission application. *Diam. Relat. Mater.* 63, 165-168 (2016).

10. Koizumi, S., Teraji, T. & Kanda, H. Phosphorus-doped chemical vapor deposition of diamond. *Diam. Relat. Mater.* 9, 935-940 (2000).

11. Williams, O. A. & Jackman, R. B. Surface conductivity on hydrogen terminated diamond. *Semicond. Sci. Technol.* 18, S34 (2003).

12. Brezeanu, M. *et al.* On-state behaviour of diamond Schottky diodes. *Diam. Relat. Mater.* 17, 736-740 (2008).

13. Abubakr, E. *et al.* Formation of low resistivity layers on singlecrystalline diamond by excimer laser irradiation. *Diam. Relat. Mater.* 95, 166-173 (2019).

14. Jubu, P. R. et al. Dispensability of the conventional Tauc's plot for accurate bandgap determination from UV-vis optical diffuse reflectance data. *Results Opt.* 9, 0-6 (2022).

15. Wen, J. H. et al. Response Time of Microfiber Temperature Sensor in Liquid Environment. *IEEE Sens. J.* 20, 6400-6407 (2020).

16. Vasil'ev, O. O., Muratov, V. B. & Duda, T. I. The study of low-temperature heat capacity of diamond: Calculation and experiment. J. *Superhard Mater.* 32, 375-382 (2010).
