# Supplementary material for: Diamond with Sp2-Sp3 composite phase for thermometry at Millikelvin temperatures
Source: Nat Commun. 2024 May 8;15:3871. doi: 10.1038/s41467-024-48137-z (PMC11079005; doi:10.1038/s41467-024-48137-z)
Supplement: Supplementary file 4 — Description of Additional Supplementary Files [file 41467_2024_48137_MOESM4_ESM.pdf]

Description of Additional Supplementary Files

Supplementary Movie 1

Description: Response time of the CPD as a temperature sensor. A sample holder containing a CPD sample was connected to a DMM7510 digital multimeter using pure silver wires in a 2-probe configuration. The sample was first allowed to equilibrate at room temperature. It was then immersed directly into liquid nitrogen. The time difference for resistance mutation represents the response time of CPD.
